# Exploring the impact of peer victimization on self-esteem in adolescents with type 1 diabetes: the power of psychological resilience

**DOI:** 10.1007/s00431-025-06203-8

**Published:** 2025-05-30

**Authors:** Aysegul Simsek, Merve Murat Mehmed Ali, Seda Er, Selmin Kose

**Affiliations:** 1 Department of Pediatric Nursing, Faculty of Health Sciences, Marmara University, Basibuyuk, Maltepe, Istanbul, Turkey; 2 Department of Psychiatric Nursing, Hamidiye Faculty of Nursing, University of Health Sciences, Istanbul, Turkey; 3 Department of Mental Health and Psychiatric Nursing, Florence Nightingale Faculty of Nursing, Istanbul University – Cerrahpaşa, Istanbul, Turkey; 4 Department of Nursing, Faculty of Health Sciences, Biruni University, Istanbul, Turkey

**Keywords:** Adolescents, Type 1 diabetes, Peer victimization, Psychological resilience, Self-esteem

## Abstract

Type 1 diabetes is common in children and adolescents. The disease causes psychosocial mismatches and makes adolescents more vulnerable to peer victimization. This study aims to determine the relationship between peer victimization and self-esteem in adolescents with type 1 diabetes and to evaluate the impact of psychological resilience in this relationship. The sample consisted of 222 adolescents with type 1 diabetes in Turkey. The research data were collected via Personal Information Form, Peer Victimization Scale, Rosenberg Self-esteem Scale-Short Form, and Adolescent Psychological Resilience Scale. Data were collected by face-to-face interviews with children. The relationships among the study variables were examined using Pearson correlation analysis, and SPSS Macro was utilized to construct the regression model and test the mediation hypotheses. Peer victimization correlated negatively with resilience (*r* = − 0.668, *p* < 0.01) and self-esteem (*r* = − 0.635, *p* < 0.01), while resilience correlated positively with self-esteem (*r* = 0.571, *p* < 0.01). Mediation analysis confirmed that peer victimization lowers self-esteem both directly and indirectly through resilience (indirect effect: − 0.012, BootLLCI = 0.019, BootULCI = − 0.005), emphasizing its protective role. *Conclusion:* Resilience plays an important role in reducing the negative effects of peer bullying on self-esteem in adolescents with type 1 diabetes. Adolescents with high resilience are better able to maintain their self-esteem even if they are bullied. However, as academic pressure and educational level increase, self-esteem may decrease more. The results underscore the necessity of interventions aimed at reducing the negative effects of peer victimization, promoting self-esteem, and enhancing psychological resilience to improve overall well-being.

**Table:** 

**What is Known:** • *Type 1 diabetes causes not only physical but also psychosocial problems in children and can expose young people to bullying behaviors.*
**What is New:** • *Psychological resilience reduces the negative effects of peer bullying. While the coping skills of adolescents exposed to bullying were weakened, those with high psychological resilience maintained their self-esteem better. Peer bullying directly and indirectly affects self-esteem negatively. Although psychological resilience mitigates some of this negative effect, self-esteem decreases significantly in adolescents exposed to bullying.* • *While age positively affects self-esteem and psychological resilience, educational level may negatively affect self-esteem. It has been observed that adolescents under academic pressure or in older age groups have more difficulty in maintaining their self-esteem.* What is significant for clinical practice? • *Diabetes management should also be addressed from a psychosocial perspective, and negative experiences (such as bullying) experienced by children should be identified and overcome by using coping mechanisms.*

**What is Known:**

• *Type 1 diabetes causes not only physical but also psychosocial problems in children and can expose young people to bullying behaviors.*

**What is New:**

• *Psychological resilience reduces the negative effects of peer bullying. While the coping skills of adolescents exposed to bullying were weakened, those with high psychological resilience maintained their self-esteem better. Peer bullying directly and indirectly affects self-esteem negatively. Although psychological resilience mitigates some of this negative effect, self-esteem decreases significantly in adolescents exposed to bullying.*

• *While age positively affects self-esteem and psychological resilience, educational level may negatively affect self-esteem. It has been observed that adolescents under academic pressure or in older age groups have more difficulty in maintaining their self-esteem.*

What is significant for clinical practice?

• *Diabetes management should also be addressed from a psychosocial perspective, and negative experiences (such as bullying) experienced by children should be identified and overcome by using coping mechanisms.*

## Introduction

Type 1 diabetes mellitus (T1DM) is the most common type of diabetes in childhood and is characterized by insulin deficiency [22, 29]. It affects approximately one quarter of the world’s population. The effects of diabetes on children include not only physical but also psychosocial aspects [2, 6]. According to the International Diabetes Federation (IDF), 1.52 million individuals under the age of 20 have diabetes worldwide and 25,759 children aged 0–19 years in Turkey have been diagnosed with T1DM [26].

The management and treatment of T1DM has been debated from many perspectives over the years [1]. Long-term management of diabetes involves various responsibilities such as insulin use, dietary modifications and lifestyle changes. This condition, which requires constant follow-up, can make social adaptation difficult, especially in adolescence, and can make individuals conspicuous among their peers [41, 49]. As children grow older and healthy growth and development continues, peer and friendship relationships become more important [9]. While these relationships are expected to always be positive, they can sometimes involve peer bullying, which can have detrimental effects on the mental and physical health of the victimized individual [3,11, 52]. This may make adolescents with T1DM more vulnerable to peer bullying. Peer bullying is the deliberate and repetitive harmful behavior of one or more children against a peer who is perceived as weaker [31, 33]. In a review, 85.7% of people with diabetes reported being victimized by their peers [4]. The psychological effects of peer victimization can negatively affect mental health, especially in adolescence. At this point, psychological resilience, which is the adaptive capacity of individuals against the difficulties they experience, is considered an important protective factor [8, 27, 15]. Psychological resilience is defined as the ability of individuals to maintain their functionality despite negative life events [28]. Psychological resilience in adolescents with T1DM may reduce the negative effects of peer bullying and contribute to the maintenance of mental well-being [14]. Self-esteem is an important indicator of psychological well-being that is closely related to psychological resilience and is defined by the individual’s feeling of self-worth and competence [28]. Peer bullying in adolescence can negatively affect self-esteem and this can lead to many problems such as weakening in social relationships, anxiety, and depression [4].

**Fig. 1:**
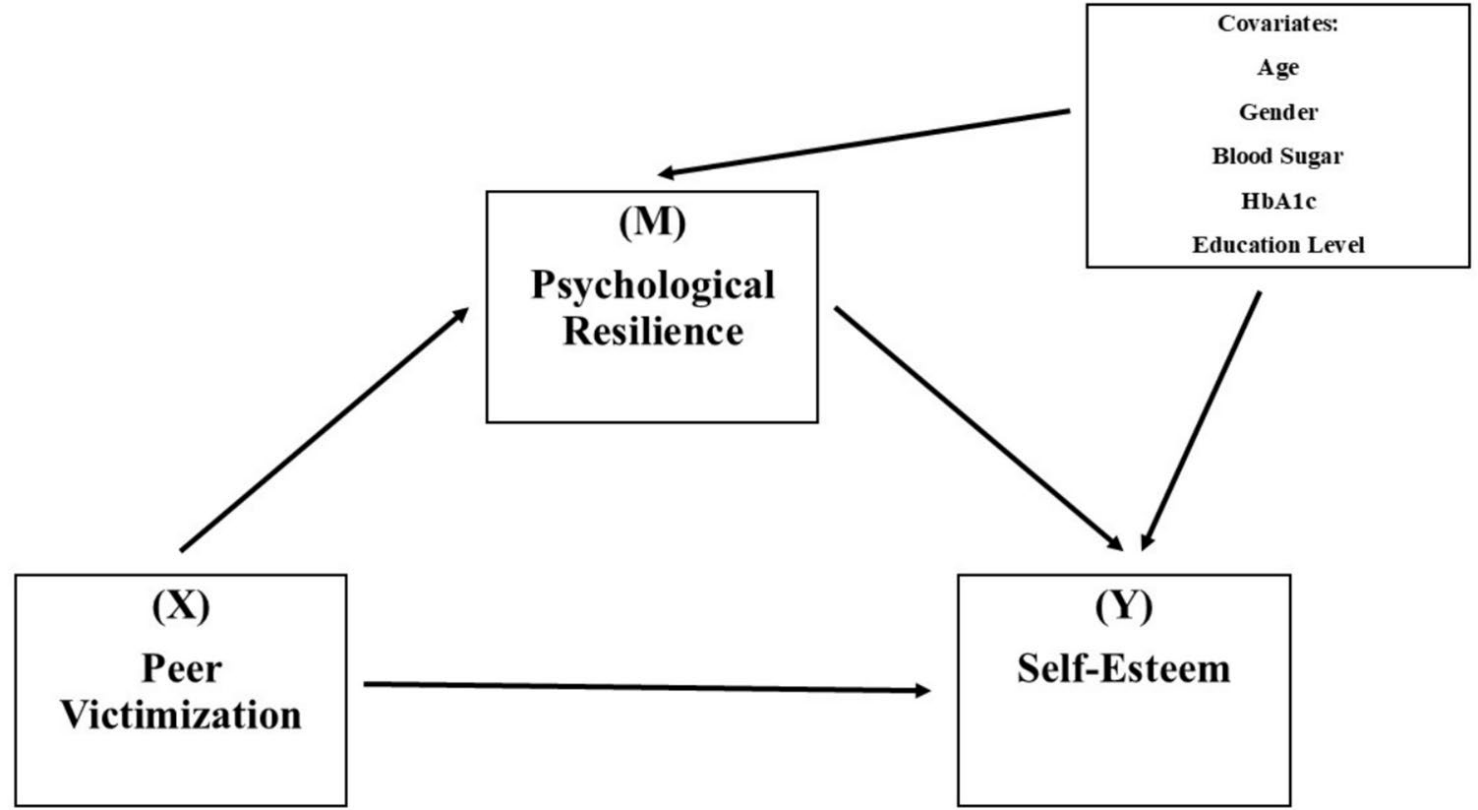
Path analysis of structure mode. The relationship between peer victimization and self-esteem in adolescents with type 1 diabetes mellitus was determined. The effect of psychological resilience on this relationship was evaluated

Although studies on adolescents’ mental health have increased in recent years, the special situation of adolescents with T1DM and their relationship with peer bullying have not been sufficiently examined. In addition, there are limited studies on the mediating role of psychological resilience in this process. In this context, the aim of this study is to examine the relationship between peer victimization and self-esteem in adolescents with T1DM and to evaluate the effect of psychological resilience on this relationship. The path analysis of the structure mode is shown in Fig. 1.

### Research questions

- H_1_: Peer victimization negatively affects psychological resilience among adolescents with T1DM.
- H_2_: Psychological resilience positively affects self-esteem among adolescents with T1DM.
- H_3_: Peer victimization negatively affects self-esteem among adolescents with T1DM.
- H_4_: Psychological resilience mediates the relationship between peer victimization and self-esteem among adolescents with T1DM.
- H_5_: Sociodemographic variables (age, gender, blood glucose level, HbA1c level, and educational status) significantly affects psychological resilience and self-esteem among adolescents with T1DM.

## Methods

### Settings

This is a descriptive and cross-sectional study.

### Participants

The study population consisted of adolescents with T1DM attending the pediatric endocrinology clinic of a state hospital in Turkey. Adolescents presenting as outpatients came to the outpatient clinic for routine control. The study was conducted between January and June 2024. The sample size study was determined based on previous research examining levels of peer victimization [49, 50]. Considering *α* = 0.05 and *β* (power) = 0.80 (small effect size: 0.20), a power analysis was conducted using G*Power (v. 3.1.9.6), which indicated that a minimum sample size of 191 participants was required. Adolescents were identified using random sampling method. The study was completed with 222 adolescents aged 11–18 years with T1DM who met the inclusion criteria.

### Inclusion and exclusion criteria

The inclusion criteria were to participate voluntarily with parental consent, to have a diagnosed psychiatric disorder (formal) or a history of psychiatric treatment (formal), and to complete all survey questions. Exclusion criteria were not speaking Turkish, presenting to the outpatient clinic with a diabetes-related health problem, and being diagnosed with T1DM for less than 1 year.

#### Data collection tools

##### Personal Information Form

The 14-question personal information form created by the researchers’ included questions about the sociodemographic characteristics of adolescents (for example, age, gender, family type, number of siblings, school information, presence of chronic diseases, body weight, height, age at diagnosis of diabetes, insulin use knowledge of diabetes among friends).

##### Peer Victimization Scale

It was developed in 2000 to assess students’ victimization experiences and adapted to Turkish in 2005 [20, 38]. The original version of the three-point Likert-type scale consists of 16 items, while the Turkish version includes 27 items ((a) victimization and (b) bullying subsections). The questions ask to what extent they experience peer victimization (items: 1a–27a) and to what extent they exhibit bullying behavior (items: 1b–27b). Scores between 0 and 54 can be obtained from the scale. A higher scale score indicates an increased tendency towards bullying behaviors. The Cronbach’s alpha coefficients for the victimization subscale were 0.93 (pre-test) and 0.90 (post-test), while for the bully subscale, they were 0.94 (pre-test) and 0.88 (post-test) [20]. In our study, Cronbach’s alpha of the scale was calculated as 0.73.

##### Rosenberg Self-esteem Scale-Short Form

It was developed in 1965 to measure self-esteem and adapted to Turkish in 1986 [13, 45]. The four-point Likert-type scale consists of ten items. There are five positive and five negative statements that are scored using the Gutmann method. Scores of 0–1 indicate high self-esteem levels, scores of 2–4 indicate moderate levels, and scores of 5–6 indicate low levels [13, 28]. A Cronbach’s alpha reliability coefficient is 0.76 and a test–retest reliability coefficient is 0.71 [13]. In our study, Cronbach’s alpha of the scale was calculated as 0.64.

##### Adolescent Psychological Resilience Scale

It was developed in 2013 to measure the level of psychological resilience [10]. It is a four-point Likert-type scale (1: not at all appropriate for me, 2: not appropriate for me, 3: appropriate for me, and 4: very appropriate for me). It consists of 29 items (10 reverse items). It has 6 sub-dimensions: family support, peer support, school support, adaptation, determination to struggle, and empathy. A score between 29 and 116 points can be obtained from the scale. Higher scores indicate higher psychological resilience. Cronbach’s alpha reliability coefficient for the whole scale is 0.87, 0.89 for family support, 0.84 for peer support, 0.81 for school support, 0.70 for adaptation, 0.67 for perseverance, and 0.61 for empathy [10]. In our study, Cronbach’s alpha was calculated as 0.74.

#### Data collection

Necessary institutional and ethics committee permissions were obtained. Then, the staff in the outpatient clinic where the study would be conducted were informed about the study. The researchers were present in the polyclinic 2 days a week (Monday and Tuesday) between 09:00–16:00. In a room in the polyclinic, the child’s parent will be interviewed. Then, the study was explained verbally and in writing to the adolescents with diabetes who met the inclusion criteria and their parents (the parent with whom they came to the control visit). Written informed consent was signed by those who agreed to participate. Then, the researchers applied the paper data collection form to the adolescents using the question-and-answer method. It took an average of 15 min per participant to complete the forms.

#### Data analysis

Data were analyzed in a computer-aided statistical program. Descriptive statistical methods (frequency, mean, and standard deviation) were used to evaluate the sociodemographic data of adolescents with diabetes. The normal distribution of the variables was also tested. The relationships among the study variables were examined using Pearson correlation analysis, and Cronbach’s alpha values were assessed to determine the reliability of the scales. SPSS Macro PROCESS (v 4.3, Hayes, 2013) was utilized to construct the regression model and test the mediation hypotheses. The PROCESS macro, which supports the analysis of multi-layered models, calculates confidence intervals (BootLLCI and BootULCI) using a bootstrap method that does not require the assumption of normal distribution—rather than relying solely on the *p*-value. If the confidence interval does not include zero, the indirect effect is considered statistically significant. Accordingly, Hayes’s (2013) Model 4 was applied in the study, with peer victimization (X) as the independent variable, self-esteem (Y) as the dependent variable, and psychological resilience (M) as the mediator variable. The significance of indirect effects was evaluated using a bootstrap method with 5000 resamples at a 95% confidence interval. The level of statistical significance was set at 0.05.

#### Ethical aspects of the study

Approval for the study was obtained from Biruni University, Non-Interventional Clinical Research Ethics Committee (Date: 22.12.2023; Decision No: 2023/85–56). Institutional permission was obtained from a training and research hospital in Istanbul (Date: 20.11.2023; Number: 1733). The study complied with the Helsinki Declaration of Human Rights.

## Results

The study was completed with 222 adolescents with a mean age of 13.94 ± 2.04 years (minimum–maximum, 12–18 years), 52.3% of whom were male. Table 1 shows the descriptive characteristics of the participants. In addition, the adolescents were diagnosed with type 1 DM at an average age of 8.21 ± 3.93 years (minimum–maximum, 1–16 years) and most of them received four insulin injections. The mean height of the participants was 153.8 ± 18.7 cm (min–max, 95–185 cm) and the mean body weight was 50.9 ± 17.8 kg (min–max, 14–98 kg). Mean last recorded blood glucose levels were 153.2 ± 58.5 mg/dL (minimum–maximum, 70–362 mg/dL) and HbA1c levels were 7.64 ± 1.6% (minimum–maximum, 5–18%) (Table 1).

**Table 1:** Sociodemographic characteristics

Characteristics		*n*	%
Gender	Female	106	47.7
	Male	116	52.3
Socioeconomical status	Income is less than expenses	33	14.9
	Income is equal to expenses	134	60.4
	Income is more than expenses	55	24.8
Family type	Nuclear family	206	92.8
	Extended family	11	5.0
	Single-parent family	5	2.3
Number of siblings	Only child	72	32.4
	1 sibling	108	48.6
	2 siblings	35	15.8
	3 and above	7	3.2
Education status	Primary school	0	0,0
	Secondary school	148	66.7
	High school	74	33.3
Academic success	Bad	13	5.9
	Average	141	63.5
	Good	68	30.6
Number of daily insulins uses	2 times	3	1.4
	3 times	12	5.4
	4 times	193	86.9
	Insulin pump	14	6.3
Other chronic diseases other than diabetes	Yes	9	4.1
	No	213	95.9
Does a first-degree relative have diabetes?	Yes	61	27.5
	No	161	72.5
Do you find family support regarding your illness sufficient?	Yes	216	97.3
	No	6	2.7
Do your friends know about your diabetes diagnosis?	Yes	210	94.6
	No	12	5.4
Do you tell your friends when you have low/high blood sugar?	Yes	172	77.5
	No	50	22.5
Total		222	100.0

*n* number, *%* percentage

Table 2 shows the distribution of Peer Victimization Scale, Rosenberg Self-esteem Scale-Short Form, and Adolescent Psychological Resilience Scale scores, while Table 3 shows the correlation analysis between the scales. Accordingly, a strong negative correlation was found between psychological resilience and peer victimization (*r* = − 0.668, *p* < 0.01), indicating that higher levels of peer victimization are associated with lower psychological resilience. This suggests that adolescents who experience victimization tend to have weaker coping skills. Additionally, resilience was positively correlated with self-esteem (*r* = 0.571, *p* < 0.01), implying that adolescents with higher resilience tend to have a stronger sense of self-worth. Furthermore, peer victimization exhibited a significant negative correlation with self-esteem (*r* = − 0.635, *p* < 0.01), indicating that increased victimization is linked to lower self-esteem.

**Table 2:** Descriptive statistics of the scales

Scales	Mean	Sd	Min	Max	Cronbach Alfa
Peer Victimization Scale	26.44	6.94	0	44	0.736
Rosenberg Self-esteem Scale-Short Form,	0.74	0.47	0.03	2.08	0.643
Adolescent Psychological Resilience Scale	57.29	12.6	20	80	0.749

*Sd* standard deviation, *min* minimum, *max* maximum

**Table 3:** Inter-scale correlation

Scales		Adolescent Psychological Resilience Scale*	Peer Victimization Scale*	Rosenberg Self-esteem Scale
Adolescent Psychological Resilience Scale	*r*	1		
	*p*			
Peer Victimization Scale	*r*	− 0.668	1	
	*p*	0.000		
Rosenberg Self-Esteem Scale	*r*	0.571	− 0.635	1
	*p*	0.000	0.000	

*r* Pearson correlation; *p < 0.001 level (2-tailed)

Table 4 summarizes the outputs from Hayes’ PROCESS macro. In Model 1, peer victimization was found to have a significant negative effect on psychological resilience (*β* = − 1.188, *p* < 0.001) and accounted for 46.4% of the variance in psychological resilience (*R*^2^ = 0.464). Among the control variables, age had a significant positive effect on psychological resilience (*β* = 1.293, *p* = 0.050), while the other sociodemographic factors did not show a significant influence on resilience. In Model 2, self-esteem was analyzed as an outcome of both peer victimization and psychological resilience. Peer victimization was found to have a negative association with self-esteem (*β* = − 0.031, *p* < 0.001), while psychological resilience was positively associated with self-esteem (*β* = 0.010, *p* < 0.001). This model also explained 46.4% of the variance in self-esteem (*R*^2^ = 0.464). Among the control variables, education status had a significant negative effect on self-esteem (*β* = − 0.268, *p* = 0.015), whereas age had a positive effect (*β* = 0.048, *p* = 0.040). However, other control variables, including gender, blood sugar level, HbA1c, and academic achievement, did not have a significant impact on self-esteem. Further analysis of the total effect of peer victimization on self-esteem revealed a significant and negative impact (*β* = − 0.043, *p* < 0.001). The direct effect of peer victimization on self-esteem was also significant and negative (*β* = − 0.031, *p* < 0.001), while resilience mediated this relationship, contributing an indirect effect of − 0.012 (BootSE = 0.003, BootLLCI = − 0.019, BootULCI = − 0.005). These findings suggest that peer victimization negatively affects self-esteem both directly and indirectly through resilience, highlighting the protective role of psychological resilience in mitigating the harmful effects of peer victimization on adolescent self-esteem.

**Table 4:** Summary of output from Hayes’ Process macro

Model 1 Summary Outcome: Adolescent Psychological Resilience Scale							*R*			*R*^2^	*F*		*p*
							.681			.464	23.088		0.000
Model							**Coeff**			**SE**	***t***		***p***
(Constant)							87.734			6.837	12.831		0.000
Peer Victimization Scale							− 1.188			0.092	− 12.891		0.000
Age							1.293			0.657	1.968		0.049
Gender							− 0.7941			1.285	− 0.617		0.537
Blood sugar level							− 0.0138			0.011	− 1.223		0.222
HbA1c level							0.206			0.384	0.537		0.591
Education							− 5.215			2.849	− 1.829		0.068
**Model 2 Summary** **Outcome: Rosenberg Self-Esteem Scale**							***R***			***R***^**2**^	***F***		***p***
							.681			.464	23.088		.000
Model							**Coeff**			**SE**	***t***		***p***
(Constant)							0.876			0.346	2.532		0.012
Peer Victimization Scale							− 0.031			0.004	− 6.688		0.000
Adolescent Psychological Resilience Scale							0.009			0.002	3.730		0.002
Age							0.047			0.025	1.887		0.040
Gender							− 0.044			0.048	− 0.901		0.368
Blood sugar level							0.002			0.004	0.433		0.664
HbA1c level							0.003			0.014	0.234		0.815
Education							− 0.267			0.1093	− 2.450		0.015
**Model 3: Peer Victimization– > Psychological Resilience – > Self-Esteem**													
**Total effect**			**Direct effect**			**Indirect effect**			**95% confidence intervals**			**Conclusion**	
**Effect**	**SE**	***t***	**Effect**	**SE**	***t***	**Effect**		**SE**	**LLCI**		**ULCI**	Partial competitive mediation	
− 0.043	0.004	− 11.850	− 0.031	0.005	− 6.688	− 0.012		0.003	− 0.019		− 0.050		

*SE* standard error, *LLCI* lower limit confidence interval, *ULCI* upper limit confidence interval

## Discussion

To our knowledge, this is the first study to examine the mediating role of psychological resilience in the relationship between peer bullying and self-esteem in adolescents with T1DM (type 1 diabetes). T1DM, one of the most common endocrinological diseases of childhood and adolescence, can lead to difficulties in psychological adjustment and increase susceptibility to peer bullying [4, 25]. Behaviors that are visible in the management of diabetes—such as blood glucose measurements, insulin injections, and dietary restrictions—can stigmatize these children among their peers and make them targets for bullying and social discrimination [32, 49–51].

In a study by Storch et al. [50], it was reported that children with diabetes were more exposed to relational bullying and had lower levels of prosocial support. However, in this study, participants reported experiencing moderate levels of peer bullying. This difference may be due to the sample characteristics. Most adolescents reported that their friends were aware of their diabetes diagnosis and blood glucose fluctuations, indicating a high level of social support and awareness. Despite this openness, participants continued to experience moderate levels of bullying, suggesting that diabetes awareness alone is not sufficient to prevent bullying.

The average height (153.8 ± 18.7) and weight (50.9 ± 17.8) values of the adolescents indicate a great diversity in physical characteristics. The absence of obvious physical characteristics that can be associated with diabetes, such as obesity, may be a factor that reduces the likelihood of being exposed to peer bullying [32, 49–51, 36].

In our study, it was observed that adolescents had medium–high academic achievement, and their blood glucose and HbA1c levels were average. It can be concluded that this has a positive effect on adolescents’ self-esteem levels. This finding contradicts previous studies suggesting that adolescents with T1DM generally have low self-esteem [5, 30, 40]. However, it is suggested that this is associated with factors such as high academic achievement, controlled blood glucose levels, and positive general health knowledge. Beyond these findings, how protective factors such as psychological resilience play a role in strengthening an individual’s self-esteem should be further included in the discussion. In particular, coping strategies (e.g., problem-solving, seeking social support) and emotional regulation mechanisms should be considered possible psychological processes underlying this mediation [12, 19]. Such mechanisms should be explored in depth in future studies.

The findings show that the psychological resilience levels of adolescents are above average, which is similar to the findings reported by Baştopcu et al. [7]. High self-esteem and strong parental relationships are protective factors that support psychological resilience [46]. At this point, it is thought that family support is not only limited to emotional support, but also improves children’s capacity to cope with stressful events, which in turn increases both resilience and self-esteem [35, 42].

Peer bullying affects not only self-esteem but also psychological resilience, which is an important factor in coping with chronic diseases. Findings have shown that high levels of bullying are associated with low psychological resilience [14, 21]. In addition, psychological resilience served as a partial mediator in the relationship between bullying and self-esteem. However, the sub-mechanisms of how this mediation works, such as how resilient individuals reinterpret the bullying experience or use their social support systems more effectively, were not elaborated in this study. Explaining such mechanisms would increase the clinical and intervention significance of the findings [17].

Research findings show that there is a positive relationship between psychological resilience and self-esteem[49–51]. However, it is important to consider how this relationship is shaped by individual characteristics (e.g., humor, optimism) and environmental resources (e.g., school climate, social support). Such factors can indirectly affect self-esteem [43].

Peer bullying is a common problem that negatively affects self-esteem in the short and long term [37, 39, 48]. However, addressing these relationships independently of the cultural context may be an incomplete assessment. The form, severity, and tolerability of bullying may vary from society to society [47]. Therefore, including cultural factors such as how individuals with diabetes are perceived, stigmatization levels, school policies, and social norms in the country where the study was conducted will contribute to the contextualization of the findings.

According to the fourth hypothesis, in this study, peer bullying had a direct negative effect on self-esteem in adolescents with T1DM and psychological resilience mediated this relationship indirectly (effect = − 0.012). Psychological resilience has been identified as a protective factor in adjustment to conflicted peer relationships [23, 24]. At this point, school-based programs or family guidance interventions that support the development of resilience can lay the foundation for the future [34, 43].

According to the fifth hypothesis, age has a positive effect on both self-esteem and psychological resilience, while educational status has a negative effect on self-esteem. However, these relationships should be examined in more detail in the context of developmental psychology. For example, cognitive abilities that develop with advancing age may increase individuals’ ability to cope with stress. At the same time, test anxiety, peer pressure, or academic expectations that come with increasing levels of education may also weaken self-esteem [16, 44, 53]. A more comprehensive consideration of these relationships could clarify areas for intervention. In addition, resilience-building strategies developed at an early age can provide long-term psychological protection [54].

### Strengths and limitations of the study

The findings of this study should be interpreted within the scope of certain limitations. It is important to note that the limited number of adolescents poses a challenge in generalizing the findings to a broader population of adolescents with T1DM. The study was conducted in a hospital environment, which may have led to limitations in adolescents’ responses. Considering this, it may be recommended to repeat the study in environments such as schools where adolescents may feel comfortable. Second, the findings are based on self-reported data, which may be influenced by adolescents’ subjective perceptions. Since bullying experiences were assessed through self-report, the extent to which adolescents experienced bullying beyond their own perceptions remains unclear. When interpreting data related to self-esteem and psychological resilience, it is important to consider that most adolescents did not report having an additional chronic illness besides diabetes, had received diabetes-related education, and perceived the support from their families as sufficient. Lastly, the cross-sectional design of this study should be acknowledged as a limitation, as it restricts the ability to infer causal relationships.

Despite its limitations, this study has several strengths. Research examining the relationships between peer victimization, self-esteem, and psychological resilience in adolescents with T1DM remains limited. In this regard, the findings of this study are believed to contribute to existing literature. Additionally, the results may provide valuable insights for research teams developing and implementing psychoeducational programs aimed at enhancing psychological resilience and self-esteem in middle and high school settings. Future research could expand on these findings by employing a longitudinal design, allowing for a more comprehensive examination of the role of psychological resilience in the relationship between peer victimization and self-esteem. Additionally, qualitative studies exploring peer victimization among adolescents with T1DM could provide deeper insights into their lived experiences. Additional studies to examine a sample with higher rates of peer victimization may shed light on this issue further. Qualitative research can also provide valuable insights into the role of internal and external factors in the relationship between peer bullying, self-esteem, and psychological resilience. Such studies may help deepen our understanding of which specific factors contribute to resilience in the face of peer victimization and self-esteem challenges. Conducting stakeholder research involving adolescents with T1DM who have experienced peer victimization, their parents, and teachers may further enhance our understanding of how self-esteem is affected and the role of psychological resilience in this process.

### Implications for practice

Experiencing peer bullying can negatively impact the functionality and social development of adolescents with chronic illnesses. Periodic screening for peer victimization among children and adolescents with chronic diseases in schools, along with an analysis of prevalence rates, may help identify at-risk students and enable early intervention. The findings of this study have important implications for physicians, mental health professionals, and childcare professionals. Ensuring the sustainability of these screenings can be supported by school nurses and professionals working in psychological counseling and guidance services. Implementing educational programs on chronic illnesses in middle and high schools could help students develop a greater awareness of their peers’ experiences, fostering understanding and empathy among adolescents. Additionally, it is recommended to design training sessions for parents and school staff on the nature of peer victimization and the resources available to help children cope with bullying. These initiatives could contribute to creating a more supportive and inclusive school environment for students with chronic conditions. Anti-bullying programs that focus on bystanders have been shown to reduce peer bullying, while interventions centered on self-esteem have been found to enhance self-esteem levels [18]. Implementing and disseminating similar programs specifically designed for adolescents with diabetes within the school context could help reduce bullying experienced by those with chronic illnesses and support the self-esteem of those who are bullied.

## Conclusion

This study highlights the critical role of psychological resilience in mitigating the negative effects of peer victimization on self-esteem among adolescents with T1DM. Overall, these results underscore the importance of resilience in reducing the harmful effects of peer victimization on self-esteem among adolescents with T1DM. Interventions aimed at strengthening psychological resilience may serve as a protective factor against the adverse effects of peer bullying.

## Data Availability

The data underlying this article will be shared on reasonable request to the corresponding author.

## References

[1] Aathira R, Jain V (2014) Advances in management of type 1 diabetes mellitus. World J Diabetes 5(5):689–696. 10.4239/wjd.v5.i5.689 25317246 10.4239/wjd.v5.i5.689 PMC4138592

[2] American Diabetes Association. (2018). 2. Classification and diagnosis of diabetes: Standards of medical care in diabetes—2018. *Diabetes care, 41*(Supplement_1), S13-S27. 10.2337/dc18-S002 29222373

[3] Anderson JR, Mayes TL, Fuller A, Hughes JL, Minhajuddin A, Trivedi MH (2022) Experiencing bullying’s impact on adolescent depression and anxiety: mediating role of adolescent resilience. J Affect Disord 310:477–483. 10.1016/j.jad.2022.04.003 35390356 10.1016/j.jad.2022.04.003

[4] Andrade CJDN, Alves CAD (2019) Relationship between bullying and type 1 diabetes mellitus in children and adolescents: a systematic review. Jornal de Pediatria 95(5):509–518. 10.1016/j.jped.2018.10.003 30391140 10.1016/j.jped.2018.10.003

[5] Artuvan Z, Yurtsever S (2020) The relationship between self-esteem and diet compliance in adolescents with type 1 diabetes. İzmir Katip Çelebi Univ Fac Health Sci J 5(1):1–5

[6] Bahar A, Tanrıverdi D (2017) Diabetes’ psychiatric and psychosocial aspects: a review. New Symposium 55(2):13–18. 10.5455/NYS.20170710101922

[7] Baştopcu Ö, Arslan S, Arslanoğlu İ (2021) Type 1 diabetes adolescents: the relationship between sleep and life quality and psychological health levels. Int Anatolia Acad Online J Health Sci 7(2):56–78

[8] Bonanno GA (2004) Loss, trauma, and human resilience: have we underestimated the human capacity to thrive after extremely aversive events? Psychol Trauma Theory Res Pract Policy 59(1):101–113. 10.1037/0003-066X.59.1.20 10.1037/0003-066X.59.1.20 14736317

[9] Brady AM, Deighton J, Stansfeld S (2021) Chronic illness in childhood and early adolescence: a longitudinal exploration of co-occurring mental illness. Dev Psychopathol 33(3):885–898. 10.1017/S0954579420000206 32362290 10.1017/S0954579420000206

[10] Bulut S, Doğan U, Altundağ Y (2013) Adolescent psychological resilience scale: validity and reliability study. Suvremena Psihologija 16(1):21–32

[11] Cheng Q, Mills-Webb K, Marquez J, Humphrey N (2025) Longitudinal relationships across bullying victimization, friendship and social support, and internalizing symptoms in early-to-middle adolescence: a developmental cascades investigation. J Youth Adolescence 1–19. 10.1007/s10964-024-02131-2 10.1007/s10964-024-02131-2 PMC12137435 39825988

[12] Compas BE, Jaser SS, Dunbar JP, Watson KH, Bettis AH, Gruhn MA, Williams E (2017) Coping and emotion regulation from childhood to early adulthood: points of convergence and divergence. Aust J Psychol 71(1):41–51. 10.1111/ajpy.12043 10.1111/ajpy.12043 PMC4038902 24895462

[13] Çuhadaroğlu F (1986) *Adölesanlarda benlik saygısı* [in Turkish] [dissertation, Hacettepe University]

[14] Deng L, Liu Y, Wang H, Yu J, Liao L (2023) Resilience mediates the effect of peer victimization on quality of life in Chongqing adolescents: from a perspective of positive childhood experiences. Front Psychol 14:1186984. 10.3389/fpsyg.2023.1186984 37564311 10.3389/fpsyg.2023.1186984 PMC10410073

[15] Earvolino-Ramirez M (2007) Resilience: a concept analysis. Nurs Forum 42(2):73–82. 10.1111/j.1744-6198.2007.00070.x 17474940 10.1111/j.1744-6198.2007.00070.x

[16] Eccles JS, Midgley C, Wigfield A, Buchanan CM, Reuman D, Flanagan C, Mac Iver D (1993) Development during adolescence: the impact of stage–environment fit on young adolescents’ experiences in schools and in families. Am Psychol 48(2):90–101. 10.1037/0003-066X.48.2.90 8442578 10.1037//0003-066x.48.2.90

[17] Fleming J, Ledogar RJ (2008) Resilience, an evolving concept: a review of literature relevant to Aboriginal research. Pimatisiwin: J Aborig Indigenous Commun Health 6(2):7–23. https://pmc.ncbi.nlm.nih.gov/articles/PMC2956753/pdf/nihms387.pdf PMC2956753 20963184

[18] Gaffney H, Ttofi MM, Farrington DP (2019) Evaluating the effectiveness of school-bullying prevention programs: an updated meta-analytical review. Aggress Violent Beh 45:111–133. 10.1016/j.avb.2018.07.001

[19] Gross JJ (2015) Emotion regulation: current status and future prospects. Psychol Inq 26(1):1–26. 10.1080/1047840X.2014.940781

[20] Gültekin Z, Sayil M (2005) A study of the reliability and validity of the Peer Victimization Scale. Turkish Psychol Articles 8(15):47–61

[21] Gündüz B, Ay İ (2021) Examining the resilience of secondary school children: peer bully and victims. Fikriyat 1(2):145–161

[22] Haller MJ, Atkinson MA, Schatz D (2005) Type 1 diabetes mellitus: etiology, presentation, and management. Pediatr Clin North Am 52(6):1553–1578. 10.1016/j.pcl.2005.07.006 16301083 10.1016/j.pcl.2005.07.006

[23] Hinduja S, Patchin JW (2017) Cultivating youth resilience to prevent bullying and cyberbullying victimization. Child Abuse Negl 73:51–62. 10.1016/j.chiabu.2017.09.010 28945996 10.1016/j.chiabu.2017.09.010

[24] Huang HW, Chen JL, Wang RH (2018) Factors associated with peer victimization among adolescents in Taiwan. J Nurs Res 26(1):52–59. 10.1097/JNR.0000000000000195 29315206 10.1097/JNR.0000000000000195

[25] Hysing, M., Elgen, I., Gillberg, C., & Lundervold, A. J. (2009). Emotional and behavioral problems in subgroups of children with chronic illness: Results from a large-scale population study. *Child: Care, Health and Development, 35*(4), 527–533. 10.1111/j.1365-2214.2009 10.1111/j.1365-2214.2009.00967.x 19323670

[26] International Diabetes Federation (2021) Diabetes Atlas (10th ed). Retrieved from https://diabetesatlas.org/atlas/tenth-edition/. Accessed 01 02 2025

[27] Karaırmak Ö (2010) Establishing the psychometric qualities of the connor-davidson resilience scale (CD-RISC) using exploratory and confirmatory factor analysis in a trauma survivor sample. Psychiatry Res 179(3):350–356 20493533 10.1016/j.psychres.2009.09.012

[28] Karaırmak Ö, Çetinkaya RS (2011) The effect of self-esteem and locus of control on resilience: the mediating role of affects. Turkish Psychol Couns Guidance J 4:30–41

[29] Katsarou A, Gudbjörnsdottir S, Rawshani A, Dabelea D, Bonifacio E, Anderson BJ, ..., Lernmark Å (2017) Type 1 diabetes mellitus. Nat Rev Disease Primers 3(1):1–17. 10.1038/nrdp.2017.16 10.1038/nrdp.2017.16 28358037

[30] Kenowitz JR, Hoogendoorn CJ, Commissariat PV, Gonzalez JS (2020) Diabetes-specific self-esteem, self-care, and glycaemic control among adolescents with type 1 diabetes. Diabet Med 37(5):760–767. 10.1111/dme.14056 31215059 10.1111/dme.14056 PMC7412989

[31] Kilicaslan F, Beyazgul B, Kuzan R, Karadag D, Koruk F, Koruk I (2023) The prevalence of peer bullying and psychiatric symptoms among high school students in southeast Turkey. Nord J Psychiatry 77(1):83–90. 10.1080/08039488.2022.2134450 36309826 10.1080/08039488.2022.2134450

[32] Köse S, Murat M (2020) Psychological symptoms in children with type-1 diabetes. Int J Caring Sci 13(1):288–293

[33] Lebrun-Harris LA, Sherman LJ, Miller B (2020) State-level prevalence of bullying victimization among children and adolescents, National Survey of Children’s Health, 2016–2017. Public Health Rep 135(3):303–309. 10.1177/0033354920912713 32243767 10.1177/0033354920912713 PMC7238709

[34] Masten AS (2014) Ordinary magic: resilience in development. Guilford Press. https://psycnet.apa.org/record/2014-24988-000. Accessed 10 Mar 2025

[35] Masten AS, Reed MGJ (2002) Resilience in development. In: Snyder CR, Lopez SJ (eds) Handbook of Positive Psychology, Oxford University Press, pp 74–88. https://www.scirp.org/reference/referencespapers?referenceid=1915259. Accessed 27 Dec 2024

[36] Moore B, Woodcock S (2017) Resilience, bullying, and mental health: factors associated with improved outcomes. Psychol Sch 54(7):689–702. 10.1002/pits.22028

[37] Mullan VM, Golm D, Juhl J, Sajid S, Brandt V (2023) The relationship between peer victimisation, self-esteem, and internalizing symptoms in adolescents: a systematic review and meta-analysis. PLoS ONE 18(3):e0282224. 10.1371/journal.pone.0282224 36989220 10.1371/journal.pone.0282224 PMC10058150

[38] Mynard H, Joseph S (2000) Development of the multidimensional peer-victimization scale. Aggress Behav: Off J Int Soc Res Aggress 26(2):169–178. 10.1002/(SICI)1098-2337(2000)26:2%3c169::AID-AB3%3e3.0.CO;2-A

[39] Overbeek G, Zeevalkink H, Vermulst A, Scholte RH (2010) Peer victimization, self-esteem, and ego resilience types in adolescents: a prospective analysis of person-context interactions. Soc Dev 19(2):270–284. 10.1111/j.1467-9507.2008.00535.x

[40] Öz R, Yılmaz HB, Akçay N (2009) The self-respect levels in children with diabetes type 1. Int J Hum Sci 6(1):330–338

[41] Palladino DK, Helgeson VS (2012) Friends or foes? a review of peer influence on self-care and glycemic control in adolescents with type 1 diabetes. J Pediatr Psychol 37(5):591–603. 10.1093/jpepsy/jss009 22460759 10.1093/jpepsy/jss009 PMC3356563

[42] Pimentel RRS, Targa T, Scardoelli MGC (2017) From diagnosis to the unknown: perceptions of parents of children and adolescents with diabetes mellitus. J Nurs UFPE/Revista de Enfermagem UFPE 11(3):1118–1126. 10.5205/reuol.10544-93905-1-RV.1103201701

[43] Ran H, Cai L, He X, Jiang L, Wang T, Yang R, ..., Xiao Y (2020) Resilience mediates the association between school bullying victimization and self-harm in Chinese adolescents. J Affect Disord 277:115–120. 10.1016/j.jad.2020.07.136 10.1016/j.jad.2020.07.136 32810666

[44] Rechenberg K, Whittemore R, Grey M (2017) Anxiety in youth with type 1 diabetes. J Pediatr Nurs 32:64–71. 10.1016/j.pedn.2016.08.007 27663096 10.1016/j.pedn.2016.08.007 PMC5743322

[45] Rosenberg M (1965) Rosenberg self-esteem scale (RSE). In Acceptance and Commitment Therapy: Measures Package, p 61. 52

[46] Sapouna M, Wolke D (2013) Resilience to bullying victimization: the role of individual, family, and peer characteristics. Child Abuse Negl 37(11):997–1006. 10.1016/j.chiabu.2013.05.009 23809169 10.1016/j.chiabu.2013.05.009

[47] Smith, P. K., Kwak, K., & Toda, Y. (Eds.). (2016). *School bullying in different cultures: Eastern and Western perspectives*. Cambridge University Press. 10.1017/CBO9781139410878

[48] Spyropoulou E, Giovazolias T (2024) Cognitive reappraisal moderates the longitudinal relationship between adolescents’ peer victimization and self-esteem: a latent interaction model. Child Psychiatry Human Dev 1–13. 10.1007/s10578-024-01688-0 10.1007/s10578-024-01688-0 PMC12628438 38446363

[49] Storch EA, Heidgerken AD, Geffken GR, Lewin AB, Ohleyer V, Freddo M, Silverstein JH (2006) Bullying, regimen self-management, and metabolic control in youth with type I diabetes. J Pediatr 148(6):784–787. 10.1016/j.jpeds.2006.01.007 16769387 10.1016/j.jpeds.2006.01.007

[50] Storch EA, Lewin AB, Silverstein JH, Heidgerken AD, Strawser MS, Baumeister A, Geffken GR (2004) Social-psychological correlates of peer victimization in children with endocrine disorders. J Pediatr 145(6):784–789. 10.1016/j.jpeds.2004.08.025 15580202 10.1016/j.jpeds.2004.08.025

[51] Storch EA, Lewin A, Silverstein JH, Heidgerken AD, Strawser MS, Baumeister A, Geffken GR (2004) Peer victimization and psychosocial adjustment in children with type 1 diabetes. Clin Pediatr 43(5):467–471. 10.1177/000992280404300508 10.1177/000992280404300508 15208752

[52] Tikkanen L, Anttila H, Ulmanen S, Pyhalto K (2024) Peer relationships and study wellbeing: upper secondary students’ experiences. Soc Psychol Educ 27:3097–3117. 10.1007/s11218-024-09942-y

[53] Yildirim PK, Yildirim E, Otrar M, Şirin A (2015) Investigating the relationship between psychological resilience and self-construal in adolescents. J Educ Sci 42(42):277–297. 10.15285/ebd.58203

[54] Zimmer-Gembeck MJ, Skinner EA (2011) The development of coping across childhood and adolescence: an integrative review and critique of research. Int J Behav Dev 35(1):1–17. 10.1177/0165025410384923

